# Use of Forehead Flap for Nasal Tip Reconstruction after Traumatic Nasal Amputation

**Published:** 2017-09

**Authors:** Mohamad Ahmadi Moghadam, Shokofeh Ahmadi Moghadam

**Affiliations:** Department of Plastic and Reconstructive Surgery, Day Hospital, Tehran, Iran

**Keywords:** Trauma, Nasal tip, Amputation, Avulsion, Forehead flap

## Abstract

Nose is one of the most important aesthetic unit of the face.Management of nasal trauma plays a significant role in the practices of the majority of facial and reconstructive surgeons. Replantation, although technically very challenging, is undoubtedly the procedure of choice following traumatic nasal amputation. Here we present an illustrative case report of the traumatic amputation of a nasal tip that was treated successfully with a paramedian forehead flap and further nasal reconstructive surgery. Use of the forehead flap was performed five hours after the occurrence of trauma and was followed by surgical repair about three weeks later. This case presents evidence that a forehead flap as a full-thickness composite graft can survive with an acceptable clinical outcome. In this particular case, the final result was satisfactory.

## INTRODUCTION

Although nasal defects created by traumatic amputation are much less common than those created by the surgical excision of regional malignancies,^1^ there is an obvious need to improve current techniques for this procedure. Among the available methods, the median forehead flap has been used for centuries and remains a workhorse flap for reconstruction of major and complex nasal defects. The technique originated almost 3000 years ago in India, where nasal amputation was a common form of social punishment for various crimes. This brought about a significant rise in the group of individuals in need of total or subtotal nasal reconstruction.^2^

In the late 18th century, Carpue found a description of this Indian technique, which gave rise to the modern era of nasal resurfacing with the use of a pedicelled forehead flap. His basic technique laid the foundation for modern nasal reconstruction for the next century. These techniques were later modified and popularized by other surgical pioneers.^3^ Here, we present the case of recent traumatic nasal tip amputation during an altercation which was successfully repaired using the paramedian forehead flap technique and further nasal reconstructive surgery.

## CASE REPORT

A 28-year-old male presented at Day General Hospital following traumatic complete nasal tip amputation after a personal altercation. The amputated tissue was detached about three hours previously at the scene of the conflict and was brought to the emergency department. It was preserved in frozen saline until the time of surgery, which occurred approximately five hours after the occurrence of the altercation. 

**Fig. 1:**
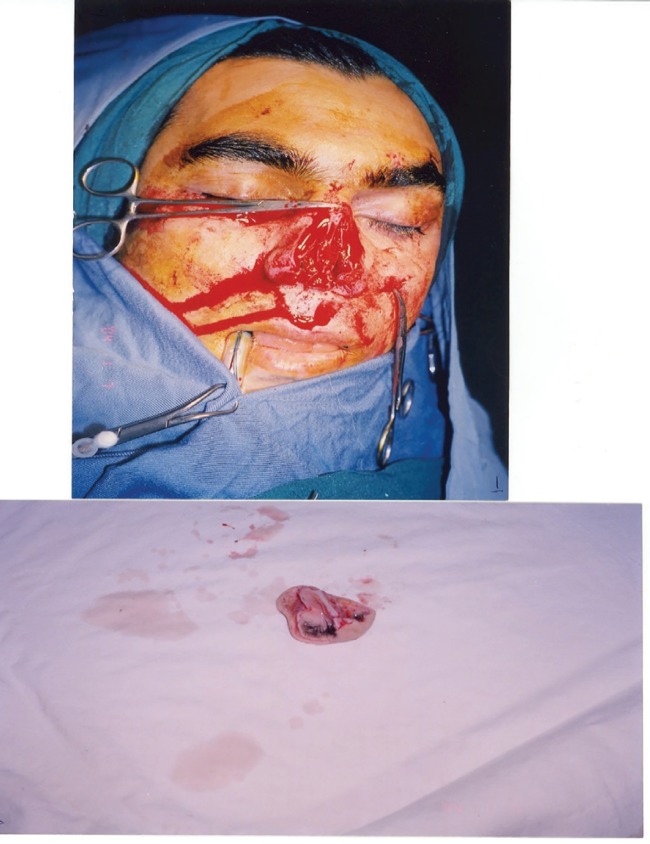
Preoperative anterior view of the patient with traumatic nasal tip amputation before surgery (above), Amputated piece of nasal tip (below

The amputated tissue measured about 2.5×2.5 cm and contained skin, alar cartilage and nasal mucosa (Figure 1). Physical examination of the area involved revealed moderate bleeding; thus, the patient was immediately treated with intravenous fluids and antibiotics, including cefazolin and tetanus globulin injections, in the emergency department. The wound was then irrigated copiously with high pressure normal saline and antiseptic solution. After a brief explanation of the procedure to the patient and his family, he was taken to the operation room for further wound debridement and surgical reparation under general anesthesia.

**Fig. 2:**
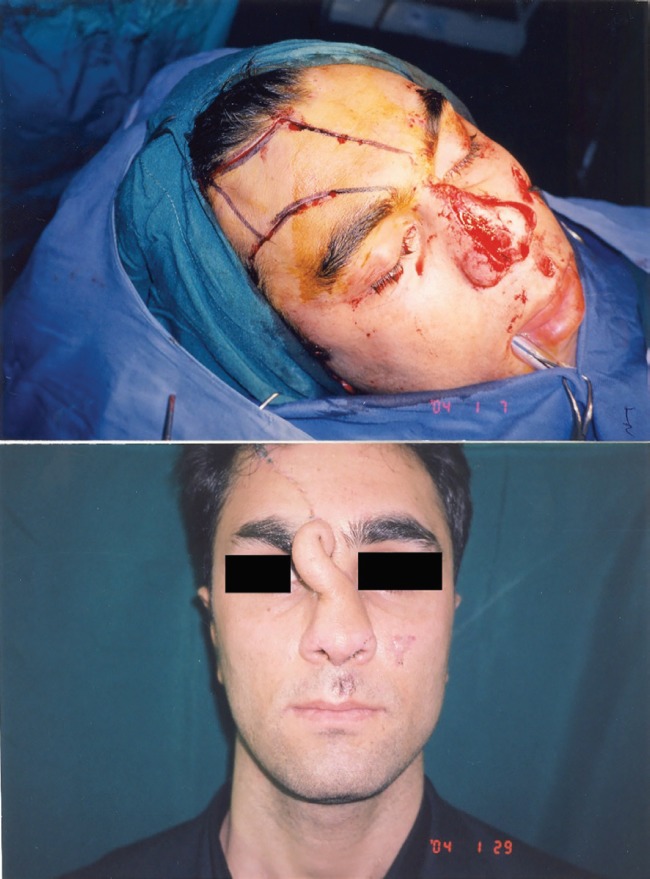
Preparing forehead flap during the first surgery (above), reconstructive forehead flap was designed and transferred over the nose defect (below

During the first operation, the skin component of the amputated tissue was resected and the residual parts, including the nasal mucosa and alar cartilage, were re-implanted in their original sites using interrupted 5-0 vicryl suture. Afterwards, the reconstructive forehead flap was designed, incised, elevated and sutured onto the nose defect. The donor site (forehead dissection) was closed in three layers (Figure 2). Post-operative care included continuous coverage with antibiotics and a prophylactic tetanus vaccination. He was discharged to home post-surgery on day 2 with an explanation of the red flag signs (fever, discharge, bleeding).

**Fig. 3:**
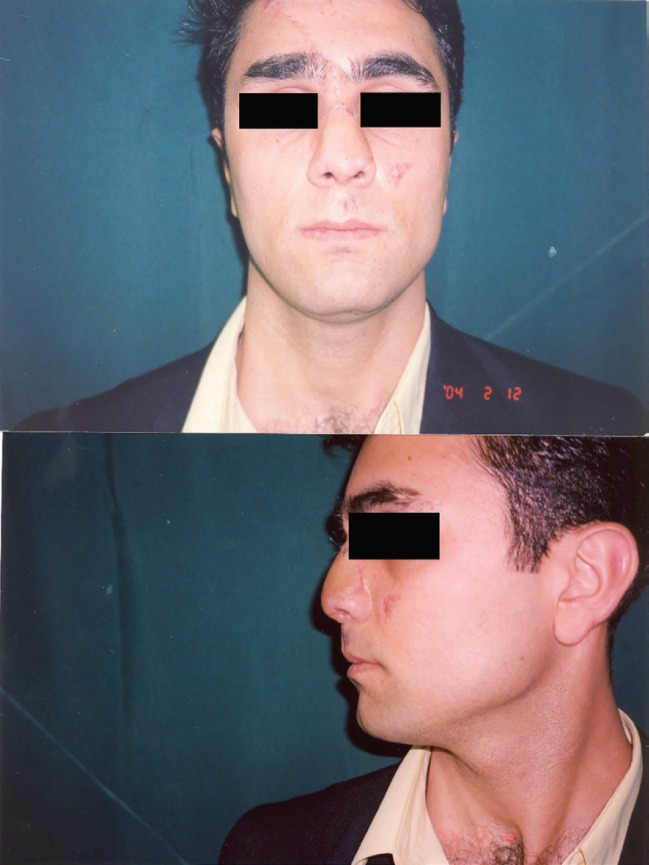
Three weeks after initial repair surgery anterior view (above), lateral view (below

Three weeks after initial repair surgery, the nasal part of the flap had become vascularized in the replanted area. At this time, extra granulation tissue of the forehead flap pedicle was excised and both the donor and recipient sites were repaired (Figure 3). 

## DISCUSSION

Although the most common etiology of nasal defects that require reconstruction is skin cancer, traumatic nasal accidents are the second most common cause. Reconstruction of extensive nasal defects including the nasal bone, septum and esthetically-defined units of the nose always present certain difficulties.^4^ Forehead flaps and replantation can be utilized for reconstruction of extensive and deep nasal defects. Clearly, the forehead flap is a reliable, valuable and well-established reconstructive option for management of extensive nasal defects.^5^ This paper presents an evaluation of the esthetic and functional results of forehead flaps for reconstruction of extensive nasal defects.

Carpue found a description of this Indian technique, giving rise to the modern era of nasal resurfacing with the use of a pedicelled forehead flap. Kazanjian advanced the development of the forehead flap by advocating primary closure of the forehead donor site. In the current case study, the ultimate deformity was smaller than the original defect and reconstruction with a midline forehead flap was successful.^6^

Documentation has advocated replantation for similar cases. Nasal replantation was first reported by Fioraventi in 1570, who described the case of a gentleman who had had his nose amputated by a Spanish soldier. In 1828, Hottacker, a German physician who attended dueling matches, similarly demonstrated the ability of cleansed, amputated nasal tissue to survive replantation. He reported the successful replantation of nasal tissue in 12 of 16 cases by simply securing the amputated tissue to the defect with tape.^7^^,^^8^ One of the greatest barriers to the success of this method was the establishment of adequate venous drainage and lack of venous outflow that resulted in graft failure due to tissue vascular congestion. The advantages of this method included the matching of the replanted tissue with the surrounding skin in terms of color and texture.^9^^,^^10^

Several authors have described the benefits of forehead flap reconstruction over other reconstructive efforts, such as the use of cheek flaps, free flaps and replantation. Nasal amputations present difficult management problems that have prompted the development of various methods for restoring function and appearance. Here, we have described the successful case of midline forehead flap and reconstructive nasal surgery in a moderately-sized nasal tip defect containing several subunits. Several advantages exist in this method. Furthermore, there is an emotional and psychological component to replacing a missing body part that is difficult to quantify, but often significant. Poor results can cause permanent deterioration of personal and social interactions on the part of the patient. 

In the present case, based on existing documentation and our own previous experience, we recommended forehead flap reconstructive surgery in the appropriated amputated nasal tissue after proper irrigation and debridement with the understanding that further reconstructive efforts would most likely be required.

## CONFLICT OF INTEREST

The authors declare no conflict of interest.
